# Exploring Young Adults' Experiences and Beliefs in Asthma Medication Management: Pilot Qualitative Study Comparing Human and Multiple AI Thematic Analysis

**DOI:** 10.2196/69892

**Published:** 2025-08-15

**Authors:** Ruth Ndarake Jeminiwa, Caroline Popielaski, Amber King

**Affiliations:** 1 Department of Pharmacy Practice, Jefferson College of Pharmacy, Thomas Jefferson University, 901 Walnut Street, Philadelphia, PA, 19107, United States, 1 2159551159; 2 Long Island Jewish Medical Center, Northwell Health, New Hyde Park, NY, United States

**Keywords:** asthma, young adults, medication management-related needs, AI, Gemini, Copilot, ChatGPT, thematic analysis

## Abstract

**Background:**

Young adults take their asthma maintenance medication 67% of the time or less. Understanding the specific needs and behaviors of young adults with asthma is essential for developing targeted interventions to improve disease self-management. Artificial intelligence (AI) has demonstrated its utility in summarizing and identifying patterns in qualitative research and may support or augment human coding efforts. However, there is pause literature to support this assertion.

**Objective:**

The objective of this study is to begin to explore the medication management-related needs of young adults with asthma via a pilot feasibility study. We aim to understand how to best assist young adults with asthma self-management and to identify potential areas where digital health interventions can provide support. We further aimed to understand the comparative outcome of human versus multiple AI platforms in performing thematic analysis.

**Methods:**

This study purposefully sampled young adults between the ages of 18 years and 29 years who had a prescription for an inhaled corticosteroid (ICS) and were either students or staff of a large metropolitan university in the northeastern United States. Semistructured interviews lasting 40 minutes on average were conducted with 4 participants via a teleconferencing application to elicit young adults’ opinions on the topic. Interviews were recorded and transcribed verbatim using Otter.ai (Otter.ai, Inc). Investigators listened to the recording to confirm the accuracy of transcriptions and to make corrections when necessary. After performing a second round of line-by-line coding, the codes were reviewed by investigators and grouped into broader, overarching themes. All investigators reviewed and discussed the final codes. Human qualitative data analyses were performed using NVivo 14 software (QSR International). After completing human analyses, the investigators performed thematic analysis with multiple AI platforms (Google Gemini, Microsoft Copilot, and OpenAI’s ChatGPT) to compare the final themes with investigator-derived themes.

**Results:**

Human analysis yielded 4 themes: support from clinicians, social support, digital self-management support, and educational support. The AI-based analysis also generated similar themes with different labels. The level of overlap on the underlying concept between humans, Gemini, Copilot, and ChatGPT was high, accounting for the fact that, although the specific labels differed, they referred to the same concept.

**Conclusions:**

Findings from our pilot exploratory study offer insights into the necessity for a holistic approach in supporting young adults with asthma. Based on the health belief model, if the identified multifaceted needs are addressed, health care systems may support medication adherence and improve health outcomes for this understudied patient population. Our pilot study also offers preliminary findings that artificial intelligence may be leveraged for successful thematic analysis of qualitative data with appropriate caution.

## Introduction

Asthma, a chronic respiratory disease, imposes a significant burden on individuals of all ages. In the United States alone, approximately 25 million people have a diagnosis of asthma, leading to missed school or work days, emergency department visits, and premature death [1,2]. According to data published by the Centers for Disease Control and Prevention, there were over 1.8 million asthma-related emergency department visits in 2019 [1]. The economic burden of asthma is substantial, estimated at US$81.9 billion per year, encompassing the cost of asthma medications, hospital visits due to asthma exacerbations, costs incurred by absenteeism, and mortality [3]. Asthma is not curable, but there are effective therapies that can significantly reduce symptoms and prevent worsening disease [4]. The Global Initiative for Asthma (GINA) guidelines recommend that all adults, adolescents, and children above the age of 5, with a diagnosis of asthma, should receive inhaled corticosteroid (ICS)-containing treatment, either as daily maintenance therapy or as as-needed ICS-formoterol for those with mild asthma [5]. Adherence to treatment regimens is crucial to avoid disease symptoms, exacerbations, and other complications in those who have more than mild asthma [5].

A particularly vulnerable group is young adults (18‐25 years), who face unique challenges such as increased independence, an optimism that may blind them to the consequences of nonadherence, and decreased familial support, all of which may contribute to poor medication adherence [6]. In fact, studies have reported that young adults take their asthma maintenance medications 67% of the time or less, which predisposes them to frequent asthma exacerbations and hospitalizations [7-9]. Young adults with uncontrolled asthma experience shame, embarrassment, anxiety, and other deleterious emotions [10]. Compared with younger children who have caregivers in charge of their asthma management, young adults are more aware and involved in their care [11]. A previous study has reported that young adults have concerns or negative beliefs about their asthma maintenance medications [12]. Understanding the specific needs and behaviors of young adults with asthma is essential for developing targeted interventions to improve disease self-management.

Digital health platforms offer a promising avenue for addressing the challenges faced by young adults. Previous studies have demonstrated the effectiveness of mobile health technologies in improving adherence to asthma maintenance medications and outcomes [12-14]. By identifying the medication management-related support needs of young adults, we can tailor digital health interventions to support medication adherence and enhance asthma outcomes for this age group. Artificial intelligence (AI) is disrupting diverse sectors, including health care. In fact, it’s been deployed in numerous capacities such as medical education, patient engagement, data analysis, and health interventions [15]. Artificial intelligence has demonstrated its utility in summarizing and identifying patterns in qualitative research and may support or augment human coding efforts; however, there is limited literature to support this assertion [16]. Comparative analyses of human versus top AI platforms in performing thematic analysis are still lacking. The objective of this study is to begin to explore the medication management-related needs of young adults with asthma via a pilot feasibility study. We aim to understand how to best assist young adults with asthma self-management and identify potential areas where digital health interventions can provide support. We further aimed to understand the comparative outcome of human versus multiple AI platforms in performing thematic analysis. In this study, pilot feasibility refers to the feasibility of our recruitment strategy to recruit young adults with asthma within our institution, as well as the practicality of using AI as a data analysis support tool.

## Methods

### Recruitment

This study purposefully sampled young adults between the ages of 18 years and 29 years who had a prescription for an ICS and were either students or staff of a large metropolitan university in the northeastern United States. Participants were recruited between October and November 2021. A study flyer was distributed across the university campus and dormitories to facilitate recruitment. Interested participants contacted the primary investigator via email and were recruited into the study if they met the eligibility criteria. 

### Ethical Considerations

Participants received a US$20 gift card as compensation for their time. This study was reviewed and approved by the primary author’s Institutional Review Board (Approval number: 21D.616). All participants provided informed consent before participation, and interview transcripts were fully anonymized before analysis. Personal identifiers—including names, locations, or any potentially identifying characteristics—were removed from transcripts to ensure participant confidentiality. In analyzing the qualitative data using AI-based tools, only deidentified and nonsensitive information was processed. No directly identifying details were input into AI models. All data were stored on secure, access-controlled servers. These procedures align with ethical principles for human participants’ research and ensure compliance with data privacy standards in the handling of qualitative health data.

### Interviews

Semistructured interviews lasting 40 minutes on average were conducted via a teleconferencing application to elicit young adults’ opinions on the topic. RJ, a pharmacy educator proficient in qualitative research, interviewed 4 participants. CP and AK are practicing pharmacists who also have varied experience with a qualitative approach. At the time of the research, CP was a student pharmacist. RJ’s previous experience conducting a randomized trial of young adults with asthma informed this qualitative research. The initial belief was that young adults with asthma may not perceive a strong link between nonadherence and negative asthma outcomes, which may have informed questions asking about their perceptions of taking daily ICSs. In addition, all 3 investigators did not have any previous knowledge of, or relationship with the participants, and no relationships were maintained after the study. Our study adopted a realist epistemological stance, seeking to understand the underlying support needs of young adults with asthma as perceived and articulated through their experiences. The interview guide contained open-ended questions related to beliefs about asthma medication, digital health, social support, and preferred sources of information (Multimedia Appendix 1). Interviews were recorded and transcribed verbatim using Otter.ai (Otter.ai, Inc). Investigators listened to the recording to confirm the accuracy of transcriptions and to make corrections when necessary.

### Data Analysis

RJ performed an initial line-by-line coding of transcripts to inform the code book. After defining the codes and discussing with CP, they both performed another round of line-by-line coding using the code book. Investigators met to discuss and resolve discrepancies. Codes were reviewed by RJ and CP and grouped into broader overarching themes. All investigators reviewed and discussed the final codes. Our analysis was guided by a realist epistemological stance to understand the medication management support needs of young adults with asthma based on their perceptions. We also maintained a reflective memo and reviewed the themes as a team to enhance the rigor and trustworthiness of our findings and to acknowledge and consider our possible influence on data analysis. Human qualitative data analyses were performed using NVivo 14 software (QSR International). Specifically, we used NVivo to perform a manual line-by-line inductive coding of the transcript. NVivo was subsequently used for the organization and grouping of codes into themes. In addition, memos were written and maintained on NVivo. After completing human analysis, the investigators performed thematic analysis with multiple AI platforms (Google Gemini, Microsoft Copilot, and OpenAI’s ChatGPT) to compare the final themes with investigator-derived themes. Specifically, OpenAI’s ChatGPT, Microsoft CoPilot, and Google Gemini were provided the following prompts: “Please read through the following transcripts and perform a thematic analysis. First, generate codes and then categorize them into emergent themes. The codes (groups of words) should be relevant to young adults’ medication-related needs. Each code should be between one to five words. I will supply the transcripts in batches. Only analyze respondents’ transcripts. Wait until I type in the phrase ‘Begin analysis’.” “Provide your findings in a table with column 1 as Themes, column 2 as Codes, column 3 as Sample Quotes and column 4, summary.” The first prompt was keyed in before supplying the transcripts. Deidentified transcripts were copied into batches and pasted in each platform. The second prompt was provided after typing in “Begin Analysis.” A final instruction was provided requesting Copilot AI to condense to 4 to 6 themes (Make it 4 to 6 themes). This instruction was not applicable to the other AI platforms, which already provided major themes alone. We assessed the overlap between the themes derived by humans and the AI platforms. The investigators repeated the AI-based analysis for each platform to verify findings. Similar to AI analysis, human thematic analysis was also focused on identifying the medication management support needs of young adults to be consistent with our primary research objective. Although the coding process was inductive, our initial readings and the development of the codebook were guided by this focus on understanding the participants’ perceived needs.

## Results

### Overview

**Figure 1.**
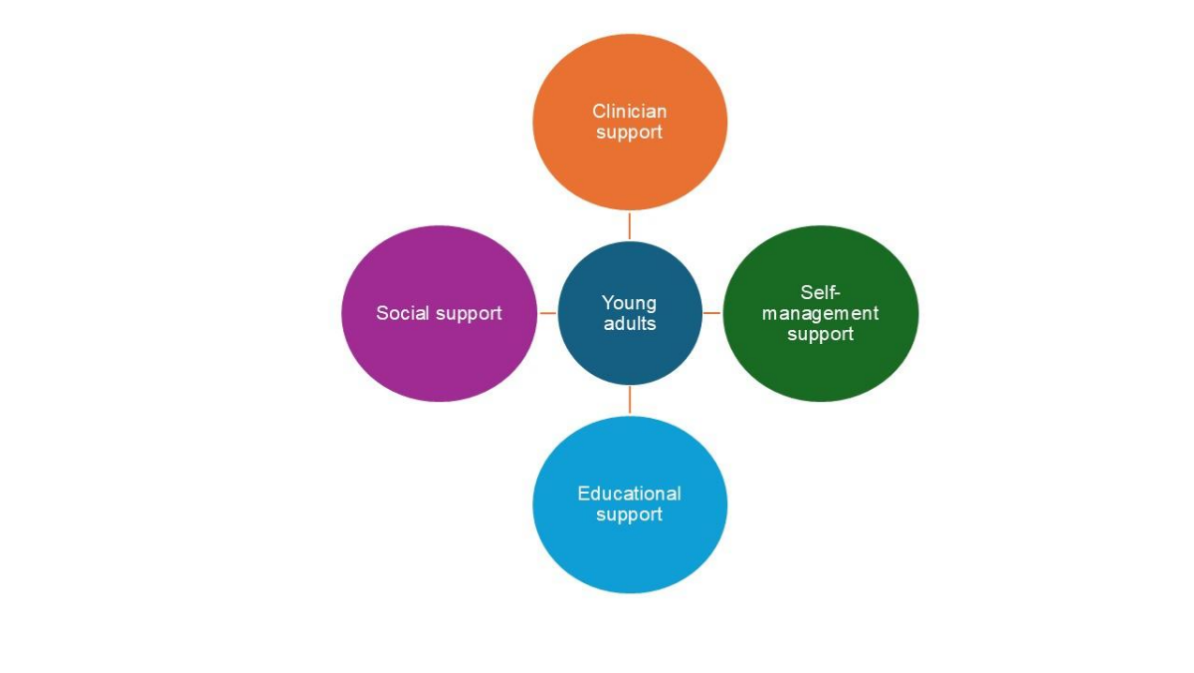
Framework of young adults’ medication support needs.

Characteristics of participants are listed in the first table (Table 1). From the investigator-based analysis, there were 4 constructed themes: support from clinicians, social support, self-management support, and educational support (Figure 1, Table 2). The AI-based analysis also generated similar themes (Table 3, Table 4, and Table 5). The level of overlap on the underlying concepts between humans, Gemini, Copilot, and ChatGPT was high, accounting for the fact that, although the specific labels differed, they referred to the same concept (Table 6). However, there were instances of divergence. For example, Gemini labeled a participant’s statement as “embarrassment,” which, while potentially related to the broader experience of managing asthma, was not identified as a central support need in the human analysis or by the other AI platforms. The team observed that repeating the analyses for each platform led to different labels for themes, varying numbers of themes, and different sample quotes. However, the results of only one iteration are reported.

**Table 1.** Characteristics of study participants.

Characteristic	Pilot sample size (n=4)
Sex, n (%)	
Female	2 (50)
Male	2 (50)
Education, n (%)	
Some college	1 (33)
Bachelor’s degree	3 (75)
Household income, n (%)	
US$25,000-US$49,000	1 (33)
US$50,000‐US$74,999	0 (0)
US$75,000-US$99,999	2 (50)
US$100,000 and above	1 (33)
Ethnicity, n (%)	
Non-Hispanic	4 (100)
Hispanic	0 (0)
Race, n (%)	
White	4 (100)
Others	0 (100)

**Table 2.** Thematic analysis result from human analysis.

Theme	Sample quotes	Summary
Support from clinicians	“One thing that would be great is if my doctor and my pharmacy had some sort of program where they would immediately send me one [medication] without me having to ask or request a new prescription”“A lot of [support from my doctor] has been with managing my flare-ups.” “I’m thankful [my allergist is] really quick about making sure they can get me refills and get me medication if I need it.”	Primary care physicians and allergists were perceived as most useful for supporting young adults. Collaboration between doctors and pharmacists is also helpful for getting medication refills on time. Support from clinicians includes prescribing medications, assessing adherence to medications, monitoring asthma symptoms, treating flare-ups, and educating on device use.
Social support	“[I received] little bit of support from my friends, but mostly my mom.” “My parents pick up my medication for me sometimes.” “My teammates know to remind me before races to take my inhaler.”	Medication-taking assistance came mainly from parents when patients were younger. Now, medication use responsibility falls almost entirely on them. Young adults also receive support from friends Social support included ensuring the availability of medications, monitoring adherence, and accompanying young adults to the doctor’s office.
Digital self-management support	“[I am] working full time. So, it gets hectic. And I think with all of that going on, just having a reminder that hey, I need to take this would help me stay consistent with it.” “Being able to track like how much of the medication you have left” “If an app could remind me to take my meds, that would be useful.” “I’d want an app connected to a doctor or health system I trust.”	Young adults may not always admit a need for help or assistance despite needing one. The biggest obstacle for young adults in managing their asthma is developing a feasible schedule that fits into their busy lifestyles. Many participants admitted a need for: a system to track inhaler use that is linked to clinicians to facilitate quicker refills, reminders to take medications daily, ensuring availability of medications, especially during trips
Educational support	“I don’t really know anything about how it makes my breathing better.” “...a reminder on how you should be taking your inhalers because I feel like I wasn’t really given [that]”	Participants were interested in learning more about asthma, device use, the different medications, and their mechanisms of action.

**Table 3.** Thematic analysis result from Google Gemini.

Theme	Code	Sample quotes	Summary
Information seeking and knowledge gaps	Doctor as source	“Mostly my allergist... So most of the information I get is through the office.” <response to a question about informational source>	Participants primarily relied on their doctors for information.
	Internet research	“I would say the internet. I think Google a lot.”	The internet served as a secondary source for information.
	Knowledge gaps	“I don’t know what the side effects are that I should be looking out for.”	Participants expressed a desire for more information about medication side effects, long-term effects, and comparisons between different medications.
Medication adherence and challenges	Medication reminders	“I think a reminder would be great. Because I think I definitely need to work on remembering to take it every day.”	Participants identified the need for reminders and external support to stay consistent with medication intake.
	Refill challenges	“They’re not very fancy with their technology... They have a phone line that’s really easy to reach, and somebody’s always answering.”	Difficulties with obtaining refills and navigating insurance coverage were common challenges.
	Daily routine	“It’s just a part of my routine, I don’t think of it as a hassle.”	Medication adherence was often tied to daily routines and habits.
Social support and family involvement	Family support	“My mom was the most important... She would always make sure I had my inhaler.”	Family members, especially parents, played a significant role in providing support, reminders, and advice regarding medication.
	Peer support	“I have a few friends that have asthma, but not, I guess not as severe as me.”	Friends were less involved in medication management, likely due to the personal nature of the condition.
	Embarrassment	“It’s at times, there’s sort of that stereotype of like, the geeky kid needs the inhaler.”	Some participants expressed feelings of embarrassment or stigma associated with using inhalers in public.
Technology and medication management	Smartphone app	“I think it could be a good idea. I would want it to be built into my calendar app.”	Participants were generally open to using smartphone apps for medication management.
	Reminders	“I think a reminder would be great. Because I think I definitely need to work on remembering to take it every day.”	Key features desired in an app included reminders, medication tracking, symptom tracking, and integration with health care providers.
	Data privacy	“I would worry about the security of that... I don’t really want to get that now.”	Concerns about data privacy and security were raised, particularly regarding sharing personal health information.
Health care provider interactions and trust	Health care provider trust	“I’ve been seeing him not too long after my diagnosis... So most of the information I get is through the office.”	Participants expressed trust in their doctors and pharmacists for medication management.
	Proactive support	“They’re just really responsive... They’re not very fancy with their technology.”	Participants desired more proactive support from health care providers, such as regular check-ins and personalized advice.
	Clear communication	“I think it’s important to have clear communication and transparency regarding medication information.” (Summarized from multiple responses)	Clear communication and transparency regarding medication information were valued by participants.

**Table 4.** Thematic analysis result output from ChatGPT.

Themes	Codes	Sample quote	Summary
Information sources and management	Trust in health care providers, family involvement	“Mostly my allergist...because I have food allergies and asthma. So, he kind of takes care of everything for me. <response to a question about informational source>”	The interviewee relies on their allergist for credible information about asthma and medications. Family members, particularly the mother, play a key role in conveying and contextualizing medical information.
Social support dynamics	Family vs friend support, shared experiences	“...mostly my mom because as primary caretaker when I was a kid... she had a lot of background for that and was very on top of, -okay, you need to take your medication...”	The interviewee receives more support from family than friends, with a shared understanding of asthma within the family, enhancing emotional support and management.
Health care professional engagement	Positive provider relationship, limited pharmacist engagement	“...my allergist, I’m thankful they’re really quick about making sure they can get me refills and get me medication if I need it.”	The interviewee has a positive relationship with their allergist, who is responsive and effective. However, they feel less engaged with pharmacists, viewing their role as limited.
Challenges with medication adherence	Forgetfulness, perceived burden	“...I think I would [benefit from additional support]... I oftentimes do forget in the morning, and I’ll remember later on in the day.”	The interviewee struggles with remembering to take daily medications and finds Symbicort more burdensome compared to the convenience of a rescue inhaler.
Technology’s role in medication management	Interest in health apps, data privacy concerns	“In general, as long as it didn’t collect too much personal information, I’d probably be pretty supportive of that.”	The interviewee shows interest in using an app for medication reminders but is concerned about data privacy and security. Desired features include reminders and side effect tracking.
Patient perceptions of medication	Medication as a chore, convenience vs long-term health	“It’s a chore. I wish that it wasn’t something I had to do... but at the same time, the rescue inhaler just feels a lot easier because I only have to remember to take it when I need it.”	The interviewee views taking Symbicort as a chore but acknowledges its effectiveness. Their preference for the convenience of a rescue inhaler reflects a common tension between immediate needs and long-term health management.
Challenges with medication adherence	Forgetfulness, perceived burden	“...my biggest concern is a breach... I think it’s definitely made me a lot more data conscious of who has what information and how that can lead to vulnerabilities.”	The interviewee struggles with remembering to take daily medications and finds Symbicort more burdensome compared to the convenience of a rescue inhaler.

**Table 5.** Thematic analysis result output from Copilot.

Themes	Codes	Sample quotes
Reliable sources of information	Mom (pediatrician), family experience, and physician check-ups	“If I had any concerns, I still ask my mom.”
Social and family support	Family support, siblings with asthma, and girlfriend’s reminders	“My girlfriend might, you know, say something like that if she notices I’m coughing a lot.”
Health care professional interaction	Medication review, prescription updates, check-ups, prescription fulfillment, mail delivery, and convenience	“My physician will certainly ask if everything is managed, everything is controlled.”
Challenges in medication adherence	Checking doses, running out of medication, traveling with medication, notification overload, and app clutter	“The biggest challenge for sure, is just planning ahead and making sure that I do have doses.”
Desired app features and health information	Built-in calendar integration, reminders, check-off feature, communication with pharmacist and doctor, symptom surveys, air quality updates, general health information, medication-specific information, and asthma health education	“I wouldn’t want a separate app, I want to use an app that I already have, probably like the calendar app or the reminder app.”

**Table 6.** Agreement with human on themes.

Human	Gemini	ChatGPT	CoPilot
Social support	Social support and family involvement	Social support dynamics	Social and family support
Clinician support	Health care provider interactions and Trust	Health care professional engagement	Health care professional interaction
Digital self-management support	Medication adherence and challenges, technology and medication management	Challenges with medication adherence, perceptions of medication, technology’s role in medication management,	Challenges in medication adherence, desired app features and health information
Educational support	Information seeking and knowledge gaps	Information sources and management	Reliable sources of information

### Themes

Social support (Social support and family involvement [Gemini]; Social support dynamics [ChatGPT]; Social and Family Support [CoPilot]): participants receive support from family members, especially their mothers. Participants acknowledged that support from family was declining, as most medication-taking responsibilities were theirs to handle. Apart from family members, participants also receive support from team members or colleagues. However, they receive more support from family than friends. Social support encompassed tangible assistance such as ensuring medication availability, monitoring medication adherence, giving medication-taking advice, and escorting young adults to doctor office visits.

Clinician support (Health care professional engagement [Gemini]; Health care professional interactions and trust [ChatGPT]; Health care professional interaction [CoPilot]): primary care physicians and allergists were the main source of clinician support for participants. This patient group desires collaboration between doctors and pharmacists to facilitate quicker prescriptions and medication refills. Participants expressed trust in their doctors and pharmacists for medication management, emphasized the need for more proactive support like regular check-ins and personalized advice, and valued clear communication and transparency regarding medication information. Clinician support encompasses prescribing medications, assessing adherence, monitoring asthma symptoms, treating exacerbations, and providing education on device use.

Digital self-management support (Medication adherence and challenges, technology and medication management [Gemini]; Challenges with medication adherence, technology and medication management; Perceptions of medication [ChatGPT]; Challenges in medication adherence, desired app features and health information [CoPilot]): participants managing asthma face challenges such as busy schedules, a need for systems to track inhaler use, and difficulties remembering daily medications. They expressed a desire for proactive support, including clinician-linked refill systems and medication reminders, especially for trips. Many find daily medication more burdensome than rescue inhalers, labeling them as a chore or something they had to do. Participants were interested in smartphone apps for medication management, but 1 person was concerned about data privacy. Desired app features include reminders, medication management, asthma attack detection, symptom tracking, built-in calendar integration, check-off feature to track adherence, air quality updates, general health information, medication-specific information, asthma health education, and integration with health care providers. External reward systems to support adherence, such as star rewards, motivated some participants to be adherent.

Educational support (Information seeking and knowledge gaps [Gemini]; Information sources and management [ChatGPT]; Reliable sources of information [CoPilot]): while the AI platforms focused on the need for trustworthy information about asthma and asthma medications, human investigators coined this theme as educational support, depicting a need for information on asthma, medication, device use, and new evidence on disease management. Participants primarily relied on their doctors for credible information about asthma and medications, with family members, particularly mothers, playing a key role in contextualizing this information. The internet served as a secondary source. There was a strong desire to receive information or education on asthma, asthma medications, device use, medication side effects, long-term medication effects, new evidence on medication outcomes, and comparison of clinical outcomes between different medications.

### Lessons Learned

There are 2 important lessons from this pilot study. First, participant recruitment via flyers alone was ineffective. Going forward, a viable collaboration with an asthma clinic or a primary care practice where young adults with asthma are seen would be a better way of recruiting patients for a similar study. Although our team made efforts to work with our asthma clinic and primary care department, the connection did not mature on time for the pilot study. Using multiple recruitment strategies may be more effective than depending on a single strategy. Second, although the findings were similar, using AI proved to be more efficient in qualitative data analysis. It took humans several months to complete the analysis compared to a few minutes for each AI platform. An AI platform could change the labels for themes, the number of themes, or sample code with different iterations of analysis.

## Discussion

### Principal Findings

This pilot feasibility study compared the thematic analysis performed by humans and multiple artificial intelligence platforms (Google Gemini, Microsoft Copilot, and ChatGPT) to assess young adults’ medication management-related needs. Findings from the study indicated that young adults need multidimensional support (social support, health care provider interaction, digital self-management support, and informational or educational support) to succeed in adherence to their daily ICSs. Apart from slight differences in the specific names of themes, humans and AI had similar results, and human analysis took a longer time to complete. For example, while Gemini labeled a theme as “Healthcare Professional Engagement,” ChatGPT labeled it as “Healthcare Professional Interaction and Trust”, CoPilot labeled it as “Healthcare Professional Interaction”, and the human labeled it “Clinician Support.” All clearly pointed to the same underlying concept of needing clinicians to communicate with patients to prescribe medications and monitor health outcomes. However, there were instances of divergence. For example, Gemini labeled a participant’s statement as “embarrassment,” which, while potentially related to the broader experience of managing asthma, was not identified as a central support need in the human analysis or by other AI platforms. In addition, ChatGPT identified “Trust in health care providers,” a theme absent in our human analysis. These divergent interpretations illustrate key differences in how AI and humans understand nuanced social context and research focus in qualitative data.

### Comparison With Previous Work

Our study demonstrated considerable overlap between thematic analysis output across the 3 AI platforms and our inductive human thematic analysis, offering preliminary support for the potential of AI supporting and augmenting human coders in qualitative studies [16]. However, there is a need for domain experts to work with AI, given the possibilities of inaccuracies without human supervision. Our study found that different iterations of analysis led to different labels for themes, different numbers of themes, and different sample quotes, suggesting a need for caution and supervision by a subject matter expert conversant with qualitative data analysis. AI will serve better as a research assistant with a likely human expert giving curated prompts and reviewing AI output for accuracy [17]. Using AI in qualitative research speeds up data analysis and leads to quicker project completions [16]. Since all 3 platforms provided similar results, selecting a specific AI for data analysis may be a matter of personal preference or what is available to the researcher. Although there has been resistance to using AI in a human-AI collaborative approach for research, AI in qualitative research could represent the future [18].

Findings from this study indicate that social support from family and friends is important to young adults. Young adults turn to these individuals for instrumental and emotional support, which may lead to satisfaction, emotional well-being, and physical functioning [19]. Our study identified potential areas for support, including participation in medication management such as reminders or check-ins, ensuring availability of medications, and accompanying a young adult for a clinician visit. Although support from family members declines compared with adolescents, young adults benefit from tangible support, encouragement, and accountability obtained from family members. Previous studies have reported that young adults involve family members in their asthma management but are cautious about burdening friends and family [20]. Based on the findings of this study, peer support is more limited and often stems from concern over seeing a young adult experience exacerbations, typically taking the form of brief questions such as, “Have you taken your meds?” Unfortunately, some young adults may not always get support from friends and family, providing an opportunity for interventions to help cultivate or leverage existing social networks to support young adults with asthma [20].

Our study found that young adults would love their health care providers to be engaged and supportive in making their care seamless and effortless. This preference is akin to the patient-centered medical home model, which promotes team-based care that is patient-centric, coordinating care across the health care system to provide seamless input and communication between providers [21]. Young adults identified allergists, primary care providers, and pharmacists as provider team members who should work together to coordinate and deliver care by prescribing medications, assessing adherence, monitoring asthma symptoms, treating exacerbations, and providing education on device use. Patient-centered medical homes show promise in delivering care that offers improved patient experience, a key aspect valued by young adults [21]. A previous study reports that emerging adults value easy access to asthma care providers, corroborating our finding on the need for support from clinicians [20].

Our study identified various opportunities for supporting asthma self-management among young adults, including medication management, refill requests, asthma symptom tracking, and exacerbation or flare-up detection before its occurrence to facilitate preventive actions. In fact, there was a strong interest in the use of smartphone apps to support asthma self-management. Similar to our study findings, reports of an earlier qualitative study indicate that young adults prefer to use technology to track asthma triggers, set reminders for medication-taking, or for education [22]. Intentional nonadherence, where individuals do not believe they need the drug, and unintentional nonadherence due to factors such as forgetfulness or changes in schedule have been reported by earlier studies investigating adherence to daily ICS in young adults with asthma [23,24]. While smartphone app reminders may address unintentional nonadherence, such as forgetfulness, there is a need for more innovative solutions to tackle intentional nonadherence. Psychological approaches such as message framing—where subtle variations in information framing can encourage people to adopt key health behaviors—hold promise in this area [12,25].

Our study highlights the need for clear, accessible information about medication use, potential side effects, and long-term benefits for young adults with asthma. Participants expressed a need for ongoing education to understand their treatment and make informed decisions, highlighting a gap that health care providers must address. Health plans and other relevant stakeholders could potentially incentivize regular educational programs that ensure that young adults with asthma are knowledgeable about their disease state, treatment options, treatment benefits, and possible side effects.

According to the health belief model, a person’s perceptions of disease severity, susceptibility to the disease, and perceived benefits and barriers to taking an action influence their health behaviors [26]. In other words, young adults with asthma may be more likely to take their daily ICSs as prescribed if they are aware of the medication, perceive themselves to be at risk of serious consequences of nonadherence, possess the self-efficacy to adhere to their medications, and experience minimal barriers to doing so. The needs identified in this study are akin to perceived barriers and may contribute to the likelihood of young adults taking their daily ICSs as prescribed, if addressed. In addition, our findings on the support needs of young adults have implications for the design of digital health applications for this patient population. For example, the expressed need for reminders, asthma attack detection, symptom tracking, check-off feature to track adherence, air quality updates, and general health information could be translated directly into app features for young adults with asthma. Mapping these features into smartphone apps may enhance the chances of improving medication adherence and asthma outcomes for this patient population, as exemplified by previous research [12,14].

The integration of AI tools into qualitative research presents both opportunities and ethical challenges [27]. In this study, we prioritized participant confidentiality by using only fully anonymized data when engaging with AI tools. Nonetheless, we acknowledge that AI platforms may raise concerns about data security and compliance with data privacy regulations [27]. We addressed these concerns by ensuring that no personally identifiable or sensitive health information was shared with AI tools. However, we recognize the broader ethical debate surrounding the use of AI in human participants’ research, particularly regarding transparency, accountability, and the potential for unintended biases in AI-generated outputs. Future work in this area should continue to explore best practices for ethical AI use in qualitative health research, including clearer regulatory guidance, institutional support for secure AI infrastructures, and increased researcher literacy on AI limitations. As this field evolves, maintaining participant trust and upholding ethical rigor will remain paramount.

### Limitations

The study provides important methodological information on how to perform qualitative analysis using AI. It compares the performance of top artificial intelligence platforms and humans on qualitative data analysis, providing helpful information for future researchers. Due to the small sample size, it was difficult to ascertain data saturation. In addition, our interpretations should be taken with caution due to the small sample size, which makes it difficult to draw meaningful conclusions. Our findings are also limited to young adults who are managing their asthma with a daily ICS. However, the aim of the pilot study was to ascertain the study’s feasibility to inform the main study and a sample size of 2-5 participants is acceptable [28]. In addition, it is important to note that our exploration of the similarities between human thematic analysis and AI is specific to the approach we used in this study, focusing on using themes related to young adults’ expressed support needs. Our findings regarding the degree of similarity observed between human thematic analysis and AI may not be transferable to other qualitative approaches that involve more in-depth interpretation, theoretical frameworks, or different analytical foci, for example, discourse analysis and narrative analysis. In addition, our focus on identifying support needs may have influenced the prompts provided to the AI platforms, potentially leading to a more directed analysis, which may not be applicable to other forms of thematic analysis and may have implications for the observed level of similarity. Furthermore, our AI comparison was limited to Google Gemini, Microsoft Copilot, and ChatGPT; findings may not apply to other AI tools. An additional limitation is the potential environmental impact of using these AI models for research.

### Conclusions

Overall, findings from our pilot exploratory study offer insight into key support needs for young adults with asthma. It underscores the necessity for a holistic approach in addressing the needs of young adults with asthma. Based on the health belief model, if identified multi-faceted needs are addressed, health care systems may support medication adherence and improve health outcomes for this understudied patient population. Our pilot study also offers preliminary findings that, for the specific inductive thematic analysis used in this research, artificial intelligence demonstrated efficiency and produced similar outputs to humans. Future studies with larger sample sizes are necessary to confirm and expand upon these initial observations. While AI shows promise for thematic analysis, critical reflections are needed regarding privacy, potential for AI over-interpretation (eg, Gemini’s “embarrassment”), environmental impact, and the non-neutral design of these platforms.

## Supplementary material

10.2196/69892 Multimedia Appendix 1 Interview guide.

## Abbreviations

AI: artificial intelligence
GINA: Global Initiative for Asthma
ICS: inhaled corticosteroid

## References

[1] (2024). Most recent national asthma data. CDC.

[2] Nunes C, Pereira AM, Morais-Almeida M (2017). Asthma costs and social impact. Asthma Res Pract.

[3] Nurmagambetov T, Kuwahara R, Garbe P (2018). The economic burden of asthma in the United States, 2008-2013. Ann Am Thorac Soc.

[4] (2024). Asthma. World Health Organization.

[5] Levy ML, Bacharier LB, Bateman E (2023). Key recommendations for primary care from the 2022 Global Initiative for Asthma (GINA) update. NPJ Prim Care Respir Med.

[6] Arnett JJ (2006). Emerging Adulthood: Understanding the New Way of Coming of Age.

[7] Withers AL, Green R (2019). Transition for adolescents and young adults with asthma. Front Pediatr.

[8] Dahlén E, Bergström A, Ödling M, Ekström S, Melén E, Kull I (2022). Non-adherence and sub-optimal treatment with asthma medications in young adults: a population-based cohort study. J Asthma.

[9] Kolmodin MacDonell K, Naar S, Gibson-Scipio W, Lam P, Secord E (2016). The Detroit young adult asthma project: pilot of a technology-based medication adherence intervention for African-American emerging adults. J Adolesc Health.

[10] Holmes LJ, Ludlow S, Fowler S, Marshall M, Lovell K (2025). Psychosocial experience of living with severe and uncontrolled asthma as a young adult: a qualitative synthesis. BMJ Open Respir Res.

[11] Kelada L, Molloy CJ, Hibbert P (2021). Child and caregiver experiences and perceptions of asthma self-management. NPJ Prim Care Respir Med.

[12] Jeminiwa R, Garza KB, Chou C, Franco-Watkins A, Fox BI (2024). Effects of framed mobile messages on beliefs, intentions, adherence, and asthma control: a randomized trial. Pharmacy (Basel).

[13] Jeminiwa R, Hohmann L, Qian J, Garza K, Hansen R, Fox BI (2019). Impact of eHealth on medication adherence among patients with asthma: A systematic review and meta-analysis. Respir Med.

[14] Unni E, Gabriel S, Ariely R (2018). A review of the use and effectiveness of digital health technologies in patients with asthma. Ann Allergy Asthma Immunol.

[15] Alhur A (2024). Redefining healthcare with artificial intelligence (AI): the contributions of ChatGPT, Gemini, and Co-pilot. Cureus.

[16] Pattyn F (2024). The value of generative AI for qualitative research: a pilot study. J Data Sci Intell Syst.

[17] Chubb LA (2023). Me and the aachines: possibilities and pitfalls of using artificial intelligence for qualitative data analysis. Int J Qual Methods.

[18] Jiang JA, Wade K, Fiesler C, Brubaker JR (2021). Supporting serendipity: opportunities and challenges for human-AI collaboration in qualitative analysis. Proc ACM Hum-Comput Interact.

[19] Lynch Milder MK, Bazier A, Ward S, Rand KL, Hirsh AT (2023). Resilience, social support, and health in emerging adults with and without chronic health conditions. Emerg Adulthood.

[20] Lee JJ, Ogini F, Hashmi M (2024). Black emerging adults with uncontrolled asthma: a qualitative study. J Allergy Clin Immunol Pract.

[21] Jackson GL, Powers BJ, Chatterjee R (2013). The patient centered medical home. A systematic review. Ann Intern Med.

[22] Murphy J, Molloy GJ, Hynes L, McSharry J (2022). Young adult preferences for digital health interventions to support adherence to inhaled corticosteroids in asthma: a qualitative study. Health Psychol Behav Med.

[23] MacDonell KK, Dailey R, Gibson-Scipio W, Wang B, Dinaj-Koci V, Bruzzese JM (2023). Exploring barriers to medication adherence among African American emerging adults with uncontrolled asthma. Health Educ Behav.

[24] MacDonell KK, Idalski Carcone A, Naar‑King S (2015). African American emerging adults’ perspectives on taking asthma controller medication: adherence in the “Age of Feeling In-Between". J Adolesc Res.

[25] Gallagher KM, Updegraff JA (2012). Health message framing effects on attitudes, intentions, and behavior: a meta-analytic review. Ann Behav Med.

[26] Alyafei A, Easton-Carr R (2024). The Health Belief Model of Behavior Change.

[27] Blessing E (2024). Regulatory compliance and ethical considerations: compliance challenges and opportunities with the integration of big data and AI. https://hal.science/hal-04972082v1/document.

[28] Aziz A, Khan N (2020). The potential uses of pilot study in qualitative research. J Res Rev Soc Sci Pak.

