# Supplementary material for: Exploring Young Adults' Experiences and Beliefs in Asthma Medication Management: Pilot Qualitative Study Comparing Human and Multiple AI Thematic Analysis
Source: JMIR Form Res. 2025 Aug 15;9:e69892. doi: 10.2196/69892 (PMC12356605; doi:10.2196/69892)
Supplement: Multimedia Appendix 1 [file formative-v9-e69892-s001.docx]

**Interview Guide**

Asthma Medication – information source

1. What or who has been the most useful source of information about asthma for you?

2. What or who has the most useful information about your asthma medication?

Asthma medication - Social Support

1. Does your friend or family member play a role in your medication-taking experience?

2. Are there other people who support your medication-taking? What is their role? How important are they?

3. What role do doctors and other health professionals play to support your medication-taking?

4. What role do pharmacists play to support your medication-taking?

5. Do you think you would benefit from additional support to take your daily medications consistently?

Beliefs about asthma medication

6. How do you feel about taking your daily inhaled corticosteroids?

7. Do you think that your daily inhaled corticosteroid (e.g., budesodine (pulmicort or symbicort), fluticasone, QVar, Flovent) works for you?

8. How important is it for you to take your daily inhaled corticosteroids (e.g., budesodine (pulmicort or symbicort), fluticasone, QVar, Flovent)? Why?

9. What motivates you to take your daily inhaled corticosteroids?

10. What are the challenges you face in taking your medications?

11. Do you have suggestions on how to address the challenge?

Smartphone App

Interviewer: We are interested in what patients think about using an app to support living with asthma.

12. What are your thoughts on using a smartphone app to support living with asthma in general?

13. How do you feel about using an app on your mobile phone to support taking your asthma medication?

14. If you were going to use an app on your phone to help you take your medications, what do you think it needs to have or do?

15. What do you think are the most effective ways to remind you to take your asthma medication? E.g., email, text message, pop-up notification from an app?

16. Have you used apps on your phone for health purposes?

17. How long do you think you can faithfully use an app to help you take your medications?

18. If you were to receive health information from an app, what content would be most helpful to help you take your medications? Is it medication-specific, general health education, or asthma health education?

19. If you were to receive health information from an app, who would you want the information to come from?

20. Would you have any concerns about getting health information (e.g., medication and self-care tips) through an app?

21. Would you have concerns about entering your health information into an app?
